# Piezoresistive, Piezocapacitive and Memcapacitive Silk Fibroin-Based Cement Mortars

**DOI:** 10.3390/s24227357

**Published:** 2024-11-18

**Authors:** Daniel A. Triana-Camacho, Antonella D’Alessandro, Silvia Bittolo Bon, Rocco Malaspina, Filippo Ubertini, Luca Valentini

**Affiliations:** 1 Department of Civil and Environmental Engineering, University of Perugia, Via G. Duranti, 06125 Perugia, Italy; danielandres.trianacamacho@unipg.it (D.A.T.-C.); antonella.dalessandro@unipg.it (A.D.); luca.valentini@unipg.it (L.V.); 2 Department of Physics and Geology, University of Perugia, Via A. Pascoli, 06123 Perugia, Italy; silvia.bittolo@gmail.com (S.B.B.); rocco.malaspina@dottorandi.unipg.it (R.M.)

**Keywords:** smart self-sensing materials, silk fibroin, cement mortars, piezoresistivity, piezocapacitance, memristive behavior, structural health monitoring

## Abstract

Water-stable proteins may offer a new field of applications in smart materials for buildings and infrastructures where hydraulic reactions are involved. In this study, cement mortars modified through water-soluble silk fibroin (SF) are proposed. Water-soluble SF obtained by redissolving SF films in phosphate buffer solution (PBS) showed the formation of a gel with the β sheet features of silk II. Electrical measurements of SF indicate that calcium ions are primarily involved in the conductivity mechanism. By exploiting the water solubility properties of silk II and Ca^2+^ ion transport phenomena as well as their trapping effect on water molecules, SF provides piezoresistive and piezocapacitive properties to cement mortars, thus enabling self-sensing of mechanical strain, which is quite attractive in structural health monitoring applications. The SF/cement-based composite introduces a capacitive gauge factor which surpasses the traditional resistive gauge factor reported in the literature by threefold. Cyclic voltammetry measurements demonstrated that the SF/cement mortars possessed memcapacitive behavior for positive potentials near +5 V, which was attributed to an interfacial charge build-up modulated by the SF concentration and the working electrode. Electrical square-biphasic excitation combined with cyclic compressive loads revealed memristive behavior during the unloading stages. These findings, along with the availability and sustainability of SF, pave the way for the design of novel multifunctional materials, particularly for applications in masonry and concrete structures.

## 1. Introduction

Water and cement are the world’s two most consumed materials. Self-sensing cement-based materials are currently at the frontier of research on structural health monitoring (SHM) and damage detection of concrete and masonry structures [1,2,3]. Self-sensing mortars and concretes can self-measure their strain conditions, thus providing key information for SHM [4]. They can be fabricated through dispersing conductive fibers or synthesizing oxides [5] or metallic particles at nanoscales [6] and then introducing these particles into construction materials, exploiting the inclusion’s capability of monitoring the mechanical strain with electrical resistance variation [7,8,9]. Carbon nanotubes, carbon nanofibers, graphene oxide and other carbon-based fillers are the best candidates nowadays for this application due to their long-term durability, reliability and good performance in cement-based pastes [10,11,12]. However, the sensing mechanism of these carbon nanomaterials relies on electron transport, which leads to significant challenges. Temperature fluctuations and other environmental factors often cause resistivity variations which exceed those induced by strain, complicating the signal processing [13,14]. Other issues related to the use of carbon-based fillers for sensing applications are associated with the difficult dispersion of the inclusions in a water-based admixture, as in cement-based ones, the occurrence of bundles or agglomerations could determine the nucleation points and weak areas of initiation of cracks or reduced strength [15,16]. The homogeneity of the material and the easiness of the production at different scales up to the real ones are crucial, essential and mandatory for the effective use of this innovative monitoring approach in construction [17]. Regenerated silk fibroin (SF) is one of most studied biomaterials which, considering the most common redissolution method of such polypeptides in water and metal ions (Ca^2+^), inspired scientists to develop soft ionic conductors [18,19,20,21]. Regenerated SF prepared by dissolution in formic acid (FA) with the addition of calcium chloride (CaCl_2_) exhibits the silk I secondary structure, which rapidly dissolves in water. In SF films prepared with a Ca^2+^/FA solution, Ca^2+^ ions can capture water from the atmosphere, coordinating and trapping water molecules via oxygen atoms [18,22]. Moreover, the secondary structural change in SF controls the sol–gel transition from a disordered state in a solution to a β sheet-rich conformation in the gel state. Thus, despite the silk I and silk II secondary conformation, it is possible to obtain SF hydrogels with a high content of water molecules.

Ionic-conductive hydrogels are perfectly suited for integration in multifunctional structures because of their liquid-like transport behavior and solid-like mechanical properties [23]. The movements of ions in a fluid material (e.g., ionic liquids) have been found to be responsible for a capacitive and resistive switching behavior [24,25]. Cement mortars which naturally combine water and cement via a hydration reaction can be used to dissolve water-soluble SF, leading to the formation of Ca ion paths. The polymorphic nature of SF indicates that the silk I structure is the key secondary structure which promotes the dissolution of SF for the regeneration of (1) water-stable and ionic conductor silk biomaterials with β sheet contents, making them processable to obtain (2) cement mortar composites. In this study, we develop water-soluble SF films containing Ca^2+^ ions which can be directed toward the electrodes and use them to fabricate SF/cement mortars (SFms). We then introduce this novel cement-based composite, highlighting its significant piezoresistive and piezocapacitive properties, which make it highly suitable for self-strain sensing in concrete and masonry structures. Finally, we demonstrate the memcapacitive state of these cement mortar composites.

## 2. Materials and Methods

### 2.1. Preparation of SF Solutions

Silk cocoons were supplied by a local farm (Fimo srl, Milano, Italy). Black phosphorous, phosphate-buffered saline (PBS), CaCl_2_, FA and NaHCO_3_ were supplied by Sigma-Aldrich. Silk solutions were prepared as reported elsewhere [26]. Briefly, the silk cocoons were degummed with NaHCO_3_ and dispersed into 5 mL FA/CaCl_2_ solution (CaCl_2_ was at a weight ratio of 70/30 with respect to the silk amount (0.70 g)). SF films were produced by leaving the SF solutions to evaporate onto Petri dishes overnight, with subsequent annealing at 40 °C for 2 h. The SF films were stirred and dissolved in PBS 1X (pH 7.4) with concentrations of 50, 100 and 200 mg/ml. SF films were produced by leaving the SF solutions to evaporate onto Petri dishes for 12 h. To monitor the sol–gel transition, the SF solutions prepared at different concentrations were cast into optically transparent bottles separately, followed by resting times of 0, 2, 24, 168 and 720 h. The sol–gel transition was determined to be when the sample vial appeared white and did not flow from the vial wall [27].

### 2.2. Preparation of the SF/Cement Mortars

**Figure 1:**
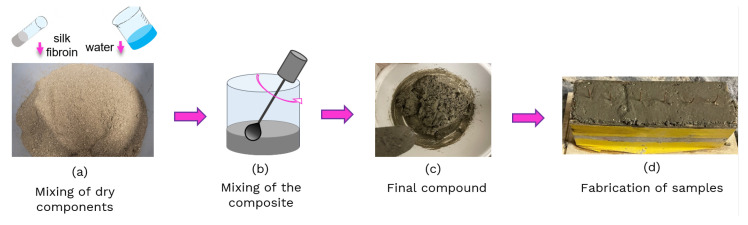
Preparation of specimens based on silk fibroin.

The SF was added to normal cement mortars constituting Portland cement type 42.5R, quarry aggregates (size: 0 ÷ 4 mm) and water. The dry components were initially mixed (Figure 1a), and then after the addition of the SF solutions at different concentrations and water, the composite sand was mixed (see Figure 1b) to obtain a composite material with suitable workability properties, as presented in Figure 1c. The compositions of the prepared samples are reported in Table 1. The sand volume was 3.5 times the cement volume, as is common for mortar composites used in the field of construction engineering [7]. A water-to-cement ratio (w/c) of 0.5 was adopted for all of the mixes.

After a mixing time of about five minutes, standard cubes with sides of 4 cm were cast with the final mixtures, and two copper wires with a diameter of 0.8 mm were embedded for 3/4 of the height at a distance of 20 mm (Figure 1d). The samples were cured at laboratory conditions for 28 days before electrical and mechanical testing.

### 2.3. Characterization of SF Solutions

The infrared spectra were obtained on the solutions in attenuated total reflection (ATR) mode using a Jasco Fourier transform spectrometer. The spectra were acquired with a resolution of 2 cm^−1^ and 300 scans per spectrum. To calculate the different contents of the secondary structures, the spectral profiles in the amide I and II regions were subjected to a curve fitting procedure, assigning a Gaussian line shape to each component with the full width at half height (FWHH) fixed at 20 cm^−1^ while allowing the other parameters to vary. The area under each component was weighted relative to the total area. Finally, the capacitance and current–voltage curves of the SF films were obtained by using a Keithley supply measurement unit (model 4200 SCS). For electrical signal extraction, the films were positioned between two Cu adhesive tapes. The relative humidity (RH) was controlled by placing the SF films in a climatic chamber at room temperature.

### 2.4. Electromechanical Characterization of SF/Cement Mortars

**Figure 2:**
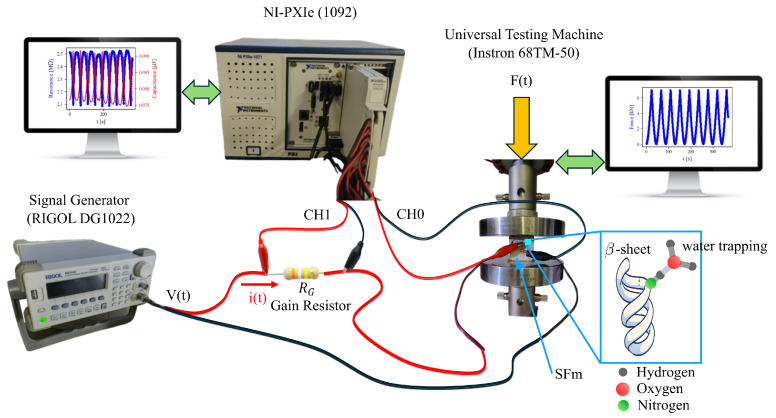
Experimental set-up to obtain electromechanical properties of SFms.

Electrical measurements were carried out using an acquisition system PXIe by National Instruments (NI). The PXIe-1092 contains two removable modules; the first is a PXIe-8840 controller card, which supports desktop operating systems, and the second is a PXIe-4313 multiplexer card capable of acquiring up to 13 analog channels. Specifically, the PXIe-4313 was employed to acquire the voltage (channel 1) from the gain resistor, which became the electrical current by dividing this voltage by the gain resistor. Simultaneously, the acquisition system measured the voltage applied to the cement-based composites through channel 0. With regard to the SFms concentrated at 50 mg/mL, 100 mg/mL and 200 mg/mL, a gain resistor of 1 MΩ was used, whereas for the mortars without SF, a gain resistor of 10 kΩ was implemented. Both the gain resistor and SFm were connected in series and powered by a signal wave generator (model: Rigol DG-1022), which provided a square voltage signal of ±10 V, hereinafter referred to as the biphasic signal, as presented Figure 2. On the other hand, cyclic voltammetry was performed on the SFms, modifying the shape and frequency in the signal generator to obtain a ±10 V triangular signal at 2.5 mHz. These frequency and amplitude values correspond to a ramp with a scan rate of 100 mV/s. Furthermore, the triangular signal was also tested at 10 mHz, which means a scan rate at 400 mV/s was used. To investigate the electromechanical coupling between the biphasic and compressive tests, these techniques were conducted at the same time. Then, the compressive tests were conducted using an Instron 68TM-50 universal testing system set to displacement-controlled mode with a load rate of 0.5 mm/min and sampled at a frequency of 50 samples per second. Ten cycles of compressive loads ranging from 0.02 kN to 7 kN were applied to all specimens, with each concentration tested in three replications to ensure reproducibility. Kapton tape was placed between the loading surfaces and specimens to prevent direct contact with the compression platens. Both the NI-PXIe system (for electrical measurements) and the universal testing machine (for mechanical measurements) were remotely operated, allowing synchronized data collection for both electrical resistance and mechanical loading. Subsequent data analysis and interpolation were performed using a custom Python script, which interpolated the timelines of the electrical and mechanical measurements. This approach enabled the reconstruction of plots which combined electrical resistance and mechanical loading data, providing a unified view of the material response under cyclic compression.

## 3. Results and Discussion

### 3.1. Physicochemical Properties of SF Dispersions

**Figure 3:**
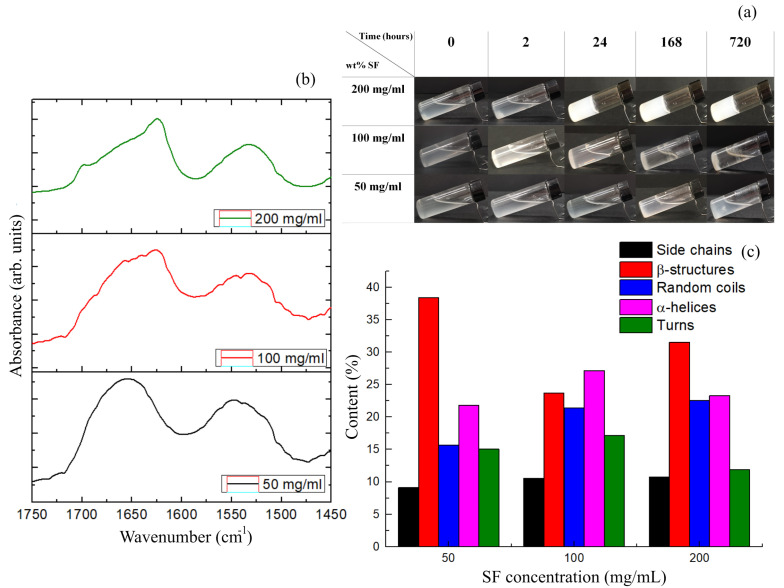
Concentration dependence of SF sol–gel transition: (**a**) dynamic optical morphology, (**b**) FTIR spectra and (**c**) relative weights of components obtained by curve–fitting procedure of FTIR spectra of SF prepared by different concentrations.

Silk fibroin-based gels are stabilized by the formation of β sheet secondary structures, which are thermodynamically more stable and function similarly to the netpoints in traditional hydrogels [28,29,30,31,32]. As the SF content in the PBS increased, the solution took on an opaque color (Figure 3a) due to light scattering caused by interaction with a heterogeneous microstructure. During the early stages (e.g., after 2 h), the SF began to form precipitates which aggregated into a gelled state, which remained stable even after 1 month. Structural changes, analyzed by ATR-FTIR and reported in Figure 1b, were due to changes in the secondary structures, particularly the bands attributed to amide I (1700–1600 cm^−1^) and amide II (1600–1500 cm^−1^). The peaks at ≈1622 cm^−1^ (amide I) and ≈1530 cm^−1^ (amide II) are characteristic of the secondary structure of silk II and are attributed to the formation of β structures [33], including silk I type II β turns, which showed absorption in the 1647–1654 cm^−1^ range. The SF films also showed absorption at 1644 cm^−1^, corresponding to a random coil structure [34]. The SF obtained from the solution with the highest SF concentration was indicative of a hydrated secondary structure, shown through an increase in random coils, with the observation of an additional peak in the amide I profile near 1622 cm^−1^, which is indicative of β sheet conformation [35] (Figure 3b,c).

**Figure 4:**
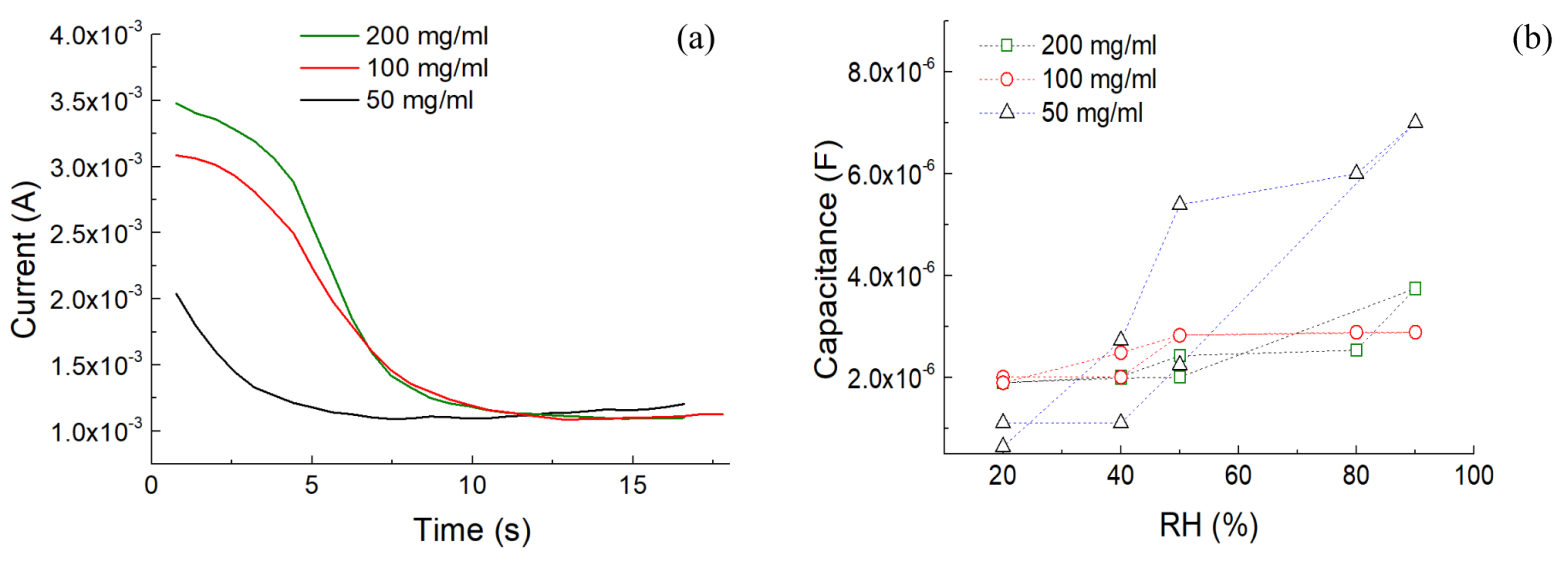
(**a**) Current transients of SF dispersions. (**b**) Capacitance–RH curve of the prepared SF dispersions with 20%→98%→50% sweeping RH.

The electrical properties of the SF solutions were studied by the change in the current versus time with an applied external voltage of 3 V (Figure 4a). The solutions were positioned between two plastic wells with adhesive Cu electrodes positioned at the bottom at a distance of 1 mm. In all cases, the current started to decrease over time. This phenomenon occurred due to ionic diffusion, which provided a change in current. The electrical current decreased at a certain time since the ions were depleted on the Cu electrode, resulting in the creation of a higher resistance value (metalization) [36]. It was found that the 200 mg/mL SF had a higher optimal water content than the 50 mg/mL SF and showed the highest ionic conductivity at the same CaCl_2_ content (Figure 4b). This behavior may have been caused by silk proteins, which inhibits the movement of ions [37]. To test the capacitive behavior, capacitance-RH measurements were performed at 10 kHz (Figure 4b). When the RH increased, the capacitance started to rise. During the reverse cycle (e.g., from 90% RH to 20% RH), the capacitance gradually decayed. The hysteretic capacitance–RH curve revealed the charge trapping/detrapping effect of water [38].

### 3.2. Characterization of SFms

In this subsection, we begin by presenting the electrical characterization of cementitious mortars added to aqueous solutions of silk fibroin prepared according to the method described in Figure 1 and whose compositions are shown in Table 1 (see the experimental section). The results of electrical characterization are presented when using both cyclic voltammetry and biphasic square signals with and without mechanical compression. Then, the non-symmetrical and nonlinear behaviors in the voltammograms were revealed, while the electromechanical measurements demonstrate that silk fibroin mortar (SFm) has the potential to be used as a strain sensor.

#### 3.2.1. Cyclic Voltammetry Study of SFms

**Figure 5:**
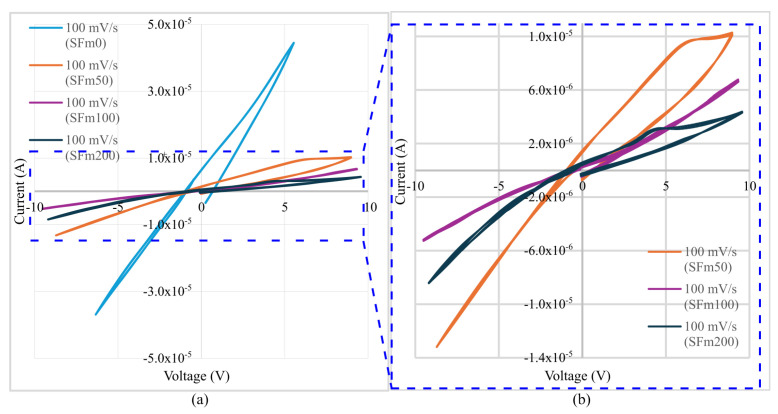
(**a**) Cyclic voltammetry of SFm specimens with SF concentrations of 0 mg/mL, 50 mg/mL, 100 mg/mL and 200 mg/mL, performed at a scan rate of 100 mV/s. (**b**) Magnification of SFm specimens with concentrations of 50 mg/mL, 100 mg/mL and 200 mg/mL.

The highest electrical current was found in the cement mortars without SF, being higher than 10 μA, and these specimens showed a quasi-linear behavior, as illustrated in Figure 5a. The affinity of SF with water molecules and surrounding ions [38] produced a fast reduction in the first days of water content being present in the SFms. In this sense, SFm100 and SFm200 held the lowest electrical current near the potential window of ±10 V.

The voltammograms in Figure 5b show the presence of oxidation peaks for positive voltages in the SFms containing 50 mg/mL (SFm50) and 200 mg/mL (SFm200) of SF. The peaks are located at 6.4 V and 4.0 V, respectively. Moreover, these oxidation peaks occurred when the voltage was dropping after it had risen to its maximum value at +10 V, indicating a capacitive discharge phenomenon instead of a Faradaic process [39,40]. On the other hand, the negative voltage region shows a linear behavior for the SFm50 specimens, but as the concentration of SF increased, the SFm100 and SFm200 curves exhibited an elbow shape, with the inflection point occurring at −2.5 V for both. Consequently, by controlling the amount of SF close to the electrodes, it was possible to create an avalanche breakdown when the SFm is reverse-biased. This result is particularly promising for the development of neural cement-based materials, as it imparts semiconductor properties to cementitious materials, opening new possibilities for integrating smart sensing and adaptive capabilities into structural engineering components [41].

#### 3.2.2. Electromechanical Properties of SFms

**Figure 6:**
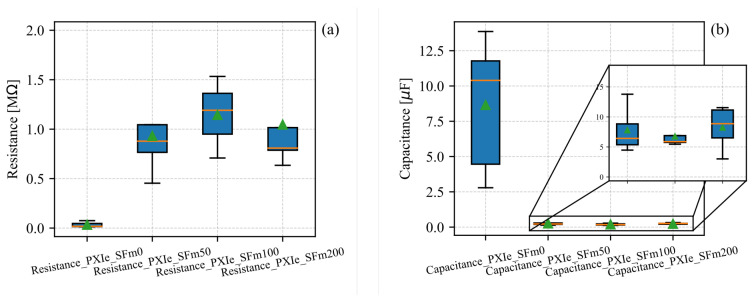
(**a**) Electrical resistance and (**b**) capacitance of SFm samples as a function of the SF concentrations.

The biphasic characterization revealed a lower electrical resistance in the reference specimens (SFm0) compared with the SFm samples, as observed in Figure 6a. This outcome was previously predicted in the voltammetric study and correlated with the water trapping effect of the SF [42]. Conversely, the capacitance increased due to the high dielectric permittivity of the water present in SFm0, as can be seen in Figure 6b. Furthermore, the specimens at an early age—5 days after the 28 day curing period—exhibited similar electrical resistance values near 1 MΩ. However, the SFm200 samples exhibited greater precision in electrical resistance compared with the other SFm samples, whereas Figure 6b shows that the SFm100 specimens had the highest precision in capacitance measurements, reaching 6.5 μF.

**Figure 7:**
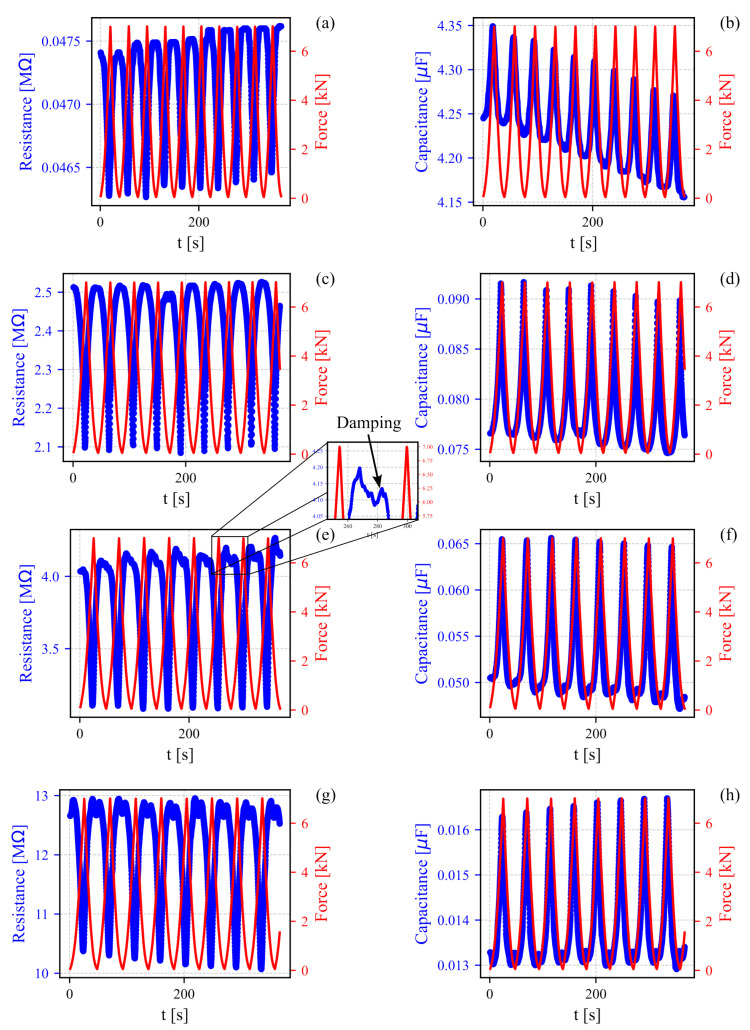
Variation in resistance and capacitance of SFm specimens with SF concentrations of (**a**,**b**) 0 mg/mL, (**c**,**d**) 50 mg/mL, (**e**,**f**) 100 mg/mL and (**g**,**h**) 200 mg/mL subjected to cyclic compressive force from 0.02 to 7 kN.

Subsequently, the results of the electromechanical characterization are shown in Figure 7. Herein, it is demonstrated how the electrical resistance and capacitance plots were adjusted because of the changes in compressive force on the SFm specimens. The SFm0 specimens (Figure 7a,b) demonstrated both piezoresistive and piezocapacitive properties while maintaining the same response profile observed during the unloading phase of the electrical tests (i.e., low resistance and high capacitance). Furthermore, the electrical resistance and capacitance signals in SFm0 were superimposed on a linear trend, which persisted in SFm50 (Figure 7c,d) but became attenuated in SFm100 and SFm200. Another notable observation during the unloading cycles at lower force values is the damping effect on the electrical resistance signal, which suggests a memory effect [40]. Damping here refers to a reduction in the amplitude of the electrical signal following a harmonic function as the strain decreased, resulting in a more stable electrical response at low strain. This stabilization implies that the electrical properties of the material remained relatively consistent in this low-strain regime after a certain setting time. This phenomenon was particularly pronounced in samples SFm100 and SFm200, as evidenced by both the piezoresistive and piezocapacitive response curves shown in Figure 7e–h.

**Figure 8:**
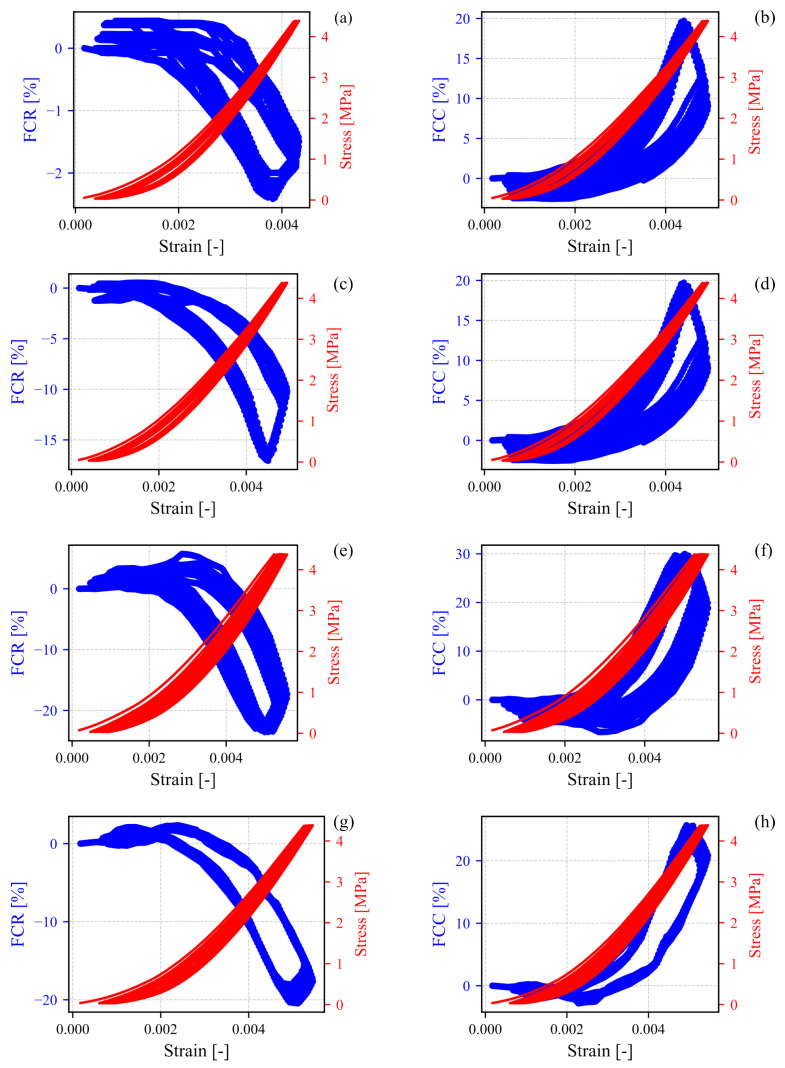
Correlation between FCR and FCC and compressive strain of SFm specimens with SF concentrations of (**a**,**b**) 0 mg/mL, (**c**,**d**) 50 mg/mL, (**e**,**f**) 100 mg/mL and (**g**,**h**) 200 mg/mL. The whole specimens were subjected to cyclic compressive force from 0.02 to 7 kN.

**Figure 9:**
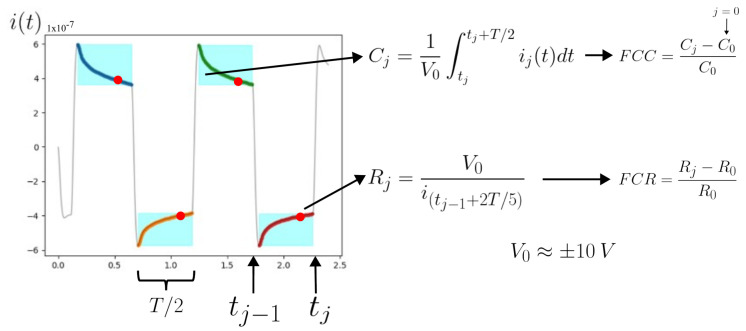
Diagram illustrating the definition of fractional change in resistance (FCR) and fractional change in capacitance (FCC) based on biphasic measurements.

Piezocapacitance is not exclusive to cementitious materials based on microfibers or nanofibers, as illustrated in Figure 8a,b and supported by the existing literature [43]. However, these piezocapacitance properties were significantly enhanced by mitigating hysteresis effects, aside from increasing the sensibility and linearity, as demonstrated in Figure 8c–h. Specifically, the fractional change in resistance (FCR) achieved a maximum magnitude of 25% for the SFm100 specimens, whereas the fractional change in capacitance (FCC) reached up to 30%. The methodology for computing the FCR and FCC is illustrated in the diagram shown in Figure 9.

It is also important to note that the linearity observed in both fractional changes in the electrical properties emerged after a strain (positive in compression) of ε=0.003, at which point the mechanical behavior also exhibited linear characteristics. Following this breakpoint, the Young’s modulus *E* and gauge factors λ were calculated, and they are presented in Table 2. *E* was calculated by extracting the linear portion of the stress–strain curves in Figure 8. These curves became approximately linear for a strain greater than 0.002, and thus this part of the plot was considered, using different cycles to compute the standard deviations, presented in Table 2. On the other hand, subscript *R* indicates the gauge factors derived from the material’s resistance, whereas subscript *C* depicts those extracted from the capacitance. The gauge factor allowed assessing the SFms’ sensitivity and reproducibility after subjecting them to cyclic strain conditions. These values were determined using the following equations:

(1) $$\lambda_{R}=\frac{FCR}{\varepsilon };\;\lambda_{C}=\frac{FCC}{\varepsilon },$$

The Young’s modulus of SFm100 presented a slight decrease of 18.7% compared with the reference specimens (SFm0) because of the SF capturing water molecules, avoiding the formation of new hydration products. On the other hand, the SFm concentrations of 50 mg/mL and 200 mg/mL exhibited an approximate 12% reduction in the Young’s modulus. Concerning the electromechanical properties, SFm100 presented the greatest enhancement in its resistive and capacitive gauge factors of 90% and 92%, respectively. These results are prominent, especially when compared with graphene/cement-based composites [44], which have shown an increasing trend with graphene concentrations below the percolation threshold, which is equal to 4% in the above reference. What is more, its theoretical gauge factor exceeds 90 [45]. In comparison with non-carbon fibers, this work presents SFm samples subjected to up to 4.5 MPa of compression, achieving a capacitive gauge factor of up to 156. For instance, Fan et al. [45] reported a maximum compressive strength of approximately 6 MPa and an experimental gauge factor near 50, while Lian et al. [8] observed a gauge factor of 107 for aligned copper-coated steel fibers concentrated at 1.5%, by using a maximum compressive strength of 20 MPa.

## 4. Conclusions

In summary, our design of silk fibroin/cement mortar composites offers a novel approach to smart sensing in cement-based materials, unlocking significant potential for SHM of masonry and concrete structures. This observation was corroborated following the physicochemical characterization of SF dispersions for analyzing the piezoelectrical response of SFm. SF exhibited an optimal water content at 45% RH, where its capacitance curves showed a steady monotonic increase. Subsequently, a decreasing trend in capacitance was observed, with an optimal point at 65% RH, potentially simulating water loss during the hydration process. Therefore, the processability of cement paste was largely influenced by the water solubility of SF, which originated from the unique conformational transitions of secondary structures. This intrinsic nature of regenerated proteins allowed us to mix SF in cement mortar and to control the ions’ mobility due to the different hydration state of SF. Thereafter, cyclic voltammetry showed SFm exhibiting a capacitive discharge process between 4.0 V and 6.4 V, which was also confirmed by the damping trend under unloading conditions. On the contrary, the bias voltage exhibited an activation threshold at −2.5 V, indicative of semiconductor behavior. In addition, the results demonstrated piezoresistive, piezocapacitive and memcapacitive properties in the SF/cement mortar composites, with the piezocapacitive GF of SFm100 being up to 16% greater than the piezoresistive GF. Nevertheless, the Young’s modulus results highlight that the enhancement in piezocapacitive properties, alongside the reduction in mechanical performance, should be addressed in future research. This is particularly important since the water retention caused by SF, if not properly handled, may hinder the hydration of cementitious composites, leading to a special focus on durability. A similar investigation is left to future work. We expect that the availability and sustainability of SF as a sensing material opens perspectives on the design of multifunctional concrete and masonry structures with self-sensing properties.

## Figures and Tables

**Table 1:** Composition of SF-based mortars.

Specimens	Cement (c)	Sand	Water (w)	SF	Ratio (w/c)
	**(g)**	**(g)**	**(g)**	**(mg/mL)**	
SFm0	172	602	86	0	0.5
SFm50	172	602	86	50	0.5
SFm100	172	602	86	100	0.5
SFm200	172	602	86	200	0.5

**Table 2:** Young’s modulus (*E*) and gauge factors, both resistive ($\lambda_{R}$) and capacitive ($\lambda_{C}$), of SFm specimens. The negative sign for the resistive GF indicates that electrical resistance decreased as the compressive force increased. Conversely, both the capacitance and compressive force increased simultaneously.

Specimen	*E* (MPa)	$-\lambda_{R}$	$\lambda_{C}$
SFm0	1455.34 ± 39.31	−13.17 ± 0.85	13.33 ± 0.75
SFm50	1281.30 ± 21.56	−92.26 ± 1.37	103.62 ± 1.46
SFm100	1182.49 ± 19.26	−131.84 ± 7.71	156.70 ± 8.12
SFm200	1272.15 ± 11.85	−115.68 ± 5.86	136.72 ± 9.73

## Data Availability

Data will be made available on request.
